# MCDA Index Tool: an interactive software to develop indices and rankings

**DOI:** 10.1007/s10669-020-09784-x

**Published:** 2020-07-16

**Authors:** Marco Cinelli, Matteo Spada, Wansub Kim, Yiwen Zhang, Peter Burgherr

**Affiliations:** 1 Future Resilient Systems (FRS), Singapore-ETH Centre (SEC), Swiss Federal Institute of Technology (ETH) Zürich, Singapore, Singapore; 2 Institute of Computing Science, Poznań University of Technology, Poznań, Poland; 3 Paul Scherrer Institut (PSI), Laboratory for Energy Systems Analysis, Villigen PSI, Switzerland

**Keywords:** Index development, Composite indicator, Software, MCDA, Normalization, Aggregation

## Abstract

A web-based software, called *MCDA Index Tool* (https://www.mcdaindex.net/), is presented in this paper. It allows developing indices and ranking alternatives, based on multiple combinations of normalization methods and aggregation functions. Given the steadily increasing importance of accounting for multiple preferences of the decision-makers and assessing the robustness of the decision recommendations, this tool is a timely instrument that can be used primarily by non-multiple criteria decision analysis (MCDA) experts to dynamically shape and evaluate their indices. The *MCDA Index Tool* allows the user to (i) input a dataset directly from spreadsheets with alternatives and indicators performance, (ii) build multiple indices by choosing several normalization methods and aggregation functions, and (iii) visualize and compare the indices’ scores and rankings to assess the robustness of the results. A novel perspective on uncertainty and sensitivity analysis of preference models offers operational solutions to assess the influence of different strategies to develop indices and visualize their results. A case study for the assessment of the energy security and sustainability implications of different global energy scenarios is used to illustrate the application of the *MCDA Index Tool*. Analysts have now access to an index development tool that supports constructive and dynamic evaluation of the stability of rankings driven by a single score while including multiple decision-makers’ and stakeholders’ preferences.

**Electronic supplementary material:**

The online version of this article (10.1007/s10669-020-09784-x) contains supplementary material, which is available to authorized users.

## Introduction

Decision-making problems are commonly based on multiple criteria and require to account for trade-offs between them before reaching a comprehensive evaluation of the alternatives under consideration (Roy 2010). This comprehensive evaluation can be reached using methods that belong to the multiple criteria decision analysis (MCDA) domain (Greco et al. 2016a). MCDA is a formal process that supports decision-making by leading the development/identification of the alternatives, the selection of the evaluation criteria (called also indicators) and the aggregation of the preferences of the stakeholders (Bouyssou et al. 2006; Cinelli 2017; Cinelli et al. 2020). There is a wide and increasing number of MCDA methods (Bisdorff et al. 2015; Greco et al. 2016a) and a main family is represented by composite indicators (CI), or indices (Diaz-Balteiro et al. 2017; El Gibari et al. 2019; Greco et al. 2019), which lead to a score of the alternatives that can then be easily ranked. Indices are used by a multitude of institutions as they can support the analysis of complex problems by means of a synthetic measure, leading to rankings and identification of trends. Some recent examples are the Covid-19-related vulnerability index by Swiss Re (SwissRe 2020), the Environmental Performance Index (Wendling et al. 2018), the Sustainable Society Index (Saisana and Philippas 2012), the Electricity Supply Resilience Index (Gasser et al. 2020), and the Global Innovation Index (Cornell University et al. 2019), to name a few. The development of indices is not a trivial task as it involves two key steps that can have crucial implications on the results. These are the normalization and the aggregation. The normalization consists in making all the indicators comparable on the same scale, while aggregation consists in defining the mathematical operator that combines the normalized indicators in the overall score/index.^1^ There are also studies which propose to develop frameworks of indicators by only using normalization, without any overarching aggregation. One example is the resilience matrix by Fox-Lent et al. (2015), where normalized indicators are used to relate the type of resilience function with the respective general management domains of any complex system (physical, information, cognitive, social). It is also notable to point out that CI development can be approached in a tiered manner. In this case, simpler models are developed first, constrained by limited resources and capital expenditures. These can then be surpassed by more complex models as more information and complexities can be accounted for in the process (Linkov et al. 2018). It has been shown that a multitude of normalization and aggregation methods exists (see OECD (2008), Jahan and Edwards (2015) and Rowley et al. (2012) for an overview) and the combination of a certain normalization and aggregation leads to a certain index.

Few studies looked at the implications of using different combinations of normalization methods and/or aggregation functions (Jahan and Edwards 2015; Narula and Reddy 2015; Pollesch and Dale 2016) and their effects on the final scores and rankings of the alternatives. The most comprehensive approach has been recently proposed by Gasser et al. (2020), where 38 combinations were used to develop the Electricity Supply Resilience Index (ESRI). ESRI is based on 12 indicators and it characterizes the resilience of 140 countries’ electricity systems. It comprehensively covers four distinct resilience functions (resist, restabilize, rebuild and reconfigure) as conceptualized in Heinimann and Hatfield (2017). This research has shown the added value of considering a multitude of perspectives of the decision makers as far as normalization of the raw data and their aggregation is concerned. In fact, the approach proposed in that article demonstrated how the robustness of the rankings can be tested. However, that research has also shown pragmatic limitations in the use of a considerable number of combinations, including the need to consistently compile the calculations and the outcome of the computations and most importantly visualize the results. In particular, that research did not conceptualize the strategies to study the variability in the output provided by the index. This is one of the research gaps tackled by this study.

This paper has two main objectives. First, it proposes an implementation strategy for variability analysis in the output of CIs, based on uncertainty and sensitivity analysis. Second, it contributes to the visualization of results by MCDA software supporting CI development. The focus is on software that allow to normalize and aggregate performances of indicators to (i) obtain a score and (ii) rank the alternatives. These contributions have been implemented in a web-based software, called MCDA Index Tool (https://www.mcdaindex.net/) that has been developed to tackle these limitations and make the methodology understandable by high-level DM and stakeholders. It allows the user to develop indices through the choice of several normalization methods and aggregation functions. The tool consists of a set of steps that guide the analyst in the development of the index starting from data loading, moving to weighting, choice of normalization methods and aggregation functions, until providing an ample set of results’ visualization.

The paper is organized as follows: Sect. 2 presents the conceptual framework to expand uncertainty and sensitivity analysis for CIs and compares software used to visualize variability of outputs in CIs. Section 3 describes the MCDA Index Tool. Section 4 provides an overview of the case study on security and sustainability of electricity supply used to show the applicability of the web-software. Section 5 presents the application of the tool to the case study. Section 6 discusses the main findings and Sect. 7 concludes by providing some recommendations for future research.

## Revisiting uncertainty and sensitivity analysis in CIs

The development of CIs is a structured process that requires a sequential set of steps to be followed (Nardo et al. 2008). The initial one consists in the creation of the conceptual framework to be evaluated, which is pivotal to obtain an understanding of the measured multidimensional phenomenon. Indicators (also called criteria by some analysts) selection and missing data management are the subsequent steps, where the variables used to quantify the target phenomenon are chosen and strategies to deal with the missing information are developed. Multivariate analysis is then required to understand the overall structure of the dataset as well as the correlations and dependencies between the indicators.^2^

At this point, the analyst has to select the preference model to aggregate the input information. Preference models represent the different philosophies of modeling in MCDA, which include (i) scoring functions, (ii) binary relations, and (iii) decision rules (Cinelli et al. 2020; Słowiński et al. 2002). CI belong to the first group (i.e., scoring functions), where the decision recommendation consists in a score for each alternative that can be used to rank the alternatives from the best to the worst one. Within such group, three choices need to be made to lead to the CI. The first consists in the selection of the normalization method, which allows transforming all the different measurement scales of the indicators in a consistent form, so that comparisons of performances among indicators are possible. The second one is the weighting of the indicators, so that the relevant weight of the indicators can be assigned to each one. The third choice is the function to aggregate the normalized performances and the weights to obtain the final score (i.e., the index). As recently shown by Gasser et al. (2020), a CI can also be calculated by combining CIs obtained from several combinations of normalization methods and aggregation functions.

Normalization is a delicate step that determines transformation of raw data into a comparable measurement scale. Several methods are available for this purpose, and they can be clustered in data-driven and expert-driven. The data-driven ones include methods like the min–max, the target and the standardized, all based on the statistical properties of the raw dataset, including minimum and maximum value and standard deviation (discussed later in Sect. 3.3), while the expert-driven ones are those that depend on the direct or indirect input of the experts/DM, for example the value theory methodology. In the latter case, raw data are normalized to a common scale by means of value functions elicited from experts and/or DMs (Geneletti and Ferretti 2015; Kadziński et al. 2020).

It is also important to acknowledge that by changing the normalization method, the relative influence of each indicator on the CI can change, as recently shown by Carrino (2017) and Gasser et al. (2019). This phenomenon is called implicit trade-offs, and it means that by changing the normalization method, the trade-offs between the indicators raw measurement scales vary. This implies that different substitution rates are needed to e.g., compensate the worsening on one indicator by improving another one.

Another recurrent distinction between normalization methods is between external and internal ones (Laurent and Hauschild 2015). External normalization is independent from the dataset and it uses reference points that do not vary if the input data changes. Internal normalization, on the contrary, provides normalized values that are dependent on the dataset. One of the main issues with internal normalization is the rank reversal problem, which means that the addition or deletion of alternatives to the set can lead to inversion in the rankings that are difficult to explain and accept by the DM. As an example, rank reversal can result in a situation where if the recommendation is that alternative A is preferred to B, and B is preferred to C, then the removal of C or the addition of a new alternative D might lead to the conclusion that B is preferred to A (Wang and Luo 2009). Until now, there is no predefined rule to select a normalization method, though each one has its own implications, which should be clarified to the DM (Carrino 2016) (see Sect. 3.3).

The aggregation stage also conceals several complexities, the main one being the level of compensation that is accepted between the different indicators (Langhans et al. 2014). In this context, compensation refers to the trade-off between the indicators, characterizing the improvement of performance needed on one indicator to offset the worsening on another indicator. One of the most common aggregation functions is the additive average, where full compensation between the indicators is assumed. This means that, independently from the actual values of the indicators, the worsening of performance on one indicator can be fully compensated by the improvement on another one (Mazziotta and Pareto 2017; Munda 2008). Several other functions have been proposed in the last decades, which allow reducing or even omitting the acceptance of compensation. Some examples are the geometric and harmonic averages (Langhans et al. 2014), the Choquet integral (Bertin et al. 2018; Grabisch and Labreuche 2016; Meyer and Ponthière 2011; Pinar et al. 2014), the outranking methods (Figueira et al. 2016), and the decision rules ones (Greco et al. 2016b). These less or non-compensatory methods are particularly useful when indicators that measure non substitutable dimensions have to be aggregated, like economic and social indicators (Bertin et al. 2018), environmental, economic and social performance (Cinelli et al. 2014; Pinar et al. 2014), strong and weak sustainability (Rowley et al. 2012), and river quality benchmarks (Reichert et al. 2015). One particular advantage of the Choquet algorithm is that it allows accounting for redundancies and positive interactions between the indicators and dimensions of a CI (Bertin et al. 2018; Pinar et al. 2014). Using interaction indices in the Choquet algorithm, it is possible, on the one hand, to assign a sort of “bonus” in the form of a reinforcement weight to the indicators which interact positively (Duarte 2018). On the other hand, for indicators that interact negatively, the Choquet algorithm permits to account for a redundancy effect so that the combined effect of these indicators on the CI can be reduced (Duarte 2018).

Once the CIs are computed, their robustness should then be studied by means of uncertainty and sensitivity analysis. Uncertainty analysis (UA) focuses on how uncertainty in inputs, such as input data and/or CI development decisions, propagates through the CI to affect outputs (Burgass et al. 2017). Sensitivity analysis (SA) studies the contribution of the individual source of uncertainty to the CI variability (Nardo et al. 2008; Saltelli et al. 2019).

The last stages of CI construction include the analysis of the results and its visualization, to make sure the outcomes are clearly and transparently communicated.

### Studying variability in the output of CIs

The assessment of the variability in the output of the CIs, being the score and ranking of the alternatives, is important to understand the stability of the provided recommendation. This variability can be studied by means of UA and SA. As described above, UA characterizes the effect of uncertainty in the CI outcome, without identifying which assumptions are primarily responsible (Saltelli et al. 2019). SA is “the study of how the uncertainty in the output of a model (numerical or otherwise) can be apportioned to different sources of uncertainty in the model input” (Saltelli and Tarantola 2002).

This paper proposes a framework, summarized in Table 1, to study output variability of CIs with UA and SA. UA and SA can be conducted on two different components of the CI, the input data, on one side, and the preference models of the CI, on the other side (Burgass et al. 2017). In this research, input data includes the indicators themselves and parameters like their weights, while preference models of the CI refer to the normalization and the aggregation stage.

**Table 1:** Proposed conceptualization of uncertainty and sensitivity analysis in CIs

Uncertainty analysis (UA)			Sensitivity analysis (SA)			
On input data (assumes a single preference model)		On preference models	On input data (assumes a single preference model)		On preference models	
Performances of indicators (e.g., uniform, normal, triangular probabilistic distributions)	Weights of indicators (e.g., systematic sampling of preference weight space)	Normalization methods + aggregation functions	Performances of indicators (e.g., change of performance, sequential exclusion)	Weights of indicators (e.g., different plausible values for the weights)	Normalization methods (including different value functions)	Aggregation functions

The most common UAs have been applied to the input data. Notable examples are the inclusion of uncertain values for the performances and/or the weights of the indicators (Dias et al. 2012; Pelissari et al. 2018). One of most common strategies is the use of stochastic input, which is conveniently modelled with probabilistic distributions (Pelissari et al. 2019). The reasons for the inclusion of uncertain input data instead of deterministic can be the presence of uncertainty in the measurement tools for the indicators’ performances, and/or the need to account for multiple weightings of the indicators themselves. Sometimes, the analysts voluntarily select uncertain input to assess how variable the results would be in case the available information is not certain or quantified variability can be foreseen, to study the stability of the decision recommendation.

An avenue of research rarely explored in UA of CI is the effect that different preference models, driven by the combined effect of normalization methods and aggregation functions can have on the final outcome. In this case, instead of looking at the variability of a single preference model (as in the case of UA on input data), the analyst can analyse the variability determined by multiple preference models. These combinations constitute the second type of UA for CI, as shown in Table 1. The rationale for such category of UA is that the analyst can consider different preferences of the DM(s) by accounting for different strategies to normalize the data and to aggregate them. The former (i.e., normalization) accounts for the desired harmonization of measurement scales, the latter (i.e., aggregation) considers the different degree of compensation that can be accepted between the indicators, ranging from a full to a null level, with gradual variations in between (Langhans et al. 2014). This type of modeling can be useful when the preferences of a group have to be included, for example. In fact, the different perspectives and value choices can result in several preference models. This proposed UA permits to jointly consider multiple preferences of the actors involved in an MCDA process and assess how variable the results can be.

As far as SA is concerned, substantial research efforts have been devoted to studying the effect of input data on the outcome (Saltelli et al. 2019). Some examples are the sequential (i.e., one-at-a-time) exclusion of the indicators, the change of the performances informed by variance estimation, or the multiple options for estimation of missing data (OECD 2008). Another avenue for SA on input data is to study the structure of the input dataset by means of statistical analysis tools, to identify for example the most influential indicators (Becker et al. 2017). When looking at the weighting, use of alternative plausible weights is a common example (Ferretti and Degioanni 2017; Triantaphyllou and Sánchez 1997). Another example is the exploration of the whole preference (weight) space, so that weights can be varied systematically to cover all possible combinations of stakeholders’ preferences (Burgherr and Spada 2014). As the SA is focused on studying the effect of each source of uncertainty, the SA on the preference models is distinguished by looking at the role of normalization methods on one side, and aggregation functions on the other side (Nardo et al. 2008). For this reason, they are presented separately in Table 1.

These UAs and SAs on the preference models are operational solutions to assess the influence of different strategies to develop the decision recommendation (i.e., scores and rankings), when using a CI.

### Visualizing variability in the output of CIs

All these UAs and SAs provide multiple scores and rankings of the alternatives, which should be visualized to discuss the variability of the outputs with the DM(s). This phase is fundamental to guarantee that the results are communicated properly and effectively (Burgass et al. 2017). For this reason, several software have been developed with multiple graphical interface capabilities to support this delicate interpretation and discussion phase. So far, limited literature has been provided on the comparison of visualization of output variability analysis in MCDA software for scoring and ranking. The main focus has been on the presentation of the methods and respective software themselves (see e.g. Weistroffer and Li (2016), Alinezhad and Khalili (2019) and Ishizaka and Nemery (2013)). A recent article by Mustajoki and Marttunen (2017) compared 23 software for supporting environmental planning processes, and focused on their capability to support the different MCDA stages. As far as analysis of results is concerned, the authors considered the presence or lack of visual graphs, overall values of the CIs (with bar charts), sensitivity analysis, *x*–*y* graphs, and written reports.

In this paper, we propose a comparison of MCDA software for scoring and ranking with a specific focus on output variability, which has not been conducted so far, according to the authors’ knowledge. The MCDA software included in this review were selected from the available compendia (Baizyldayeva et al. 2013; Ishizaka and Nemery 2013; Mustajoki and Marttunen 2017; Vassilev et al. 2005; Weistroffer and Li 2016). The search also incorporated software listed in the dedicated web pages of MCDA societies (EWG-MCDA 2020; MCDM 2020; Oleson 2016). To ensure comparability between the software results, the inclusion of software had to be limited to those that use UA and/or SA using scoring functions based on normalization. Lastly, the focus for type of software was on users labelled as “target 1” users by Mustajoki and Marttunen (2017). These are experts in a specific application domain (e.g., environmental management, energy modeling, health technology assessment, urban planning, econometrics), who want to use MCDA methods to facilitate the decision making process and enhance the visualization of the results for DMs/stakeholders. For this reason, some advanced software that require programming skills or solid knowledge of the building blocks of each method have not been included (e.g., Analytica (Lumina 2020), Diviz (Meyer and Bigaret 2012)). Eleven (11) software (including the one proposed in this paper) did fit with the inclusion requirements and they are presented in Tables 2 and 3. It is possible that some software might have been omitted in the search, but for achieving the objective of providing an overview of the main strategies used to conduct and visualize outputs variability in MCDA software for scoring and ranking, the authors think that the selected set of software was broad and diversified enough.

**Table 2:** Available uncertainty and sensitivity analysis in the selected MCDA software

Software	References	Normalization	Aggregation	Uncertainty analysis (UA)			Sensitivity analysis (SA)			
				On input data (assumes a single preference model)		On preference models	On input data (assumes a single preference model)		On preference models	
				Performances of indicators	Weights of indicators	Normalization methods + aggregation functions	Performances of indicators	Weights of indicators	Normalization methods	Aggregation functions
MCDA Index Tool	This paper	8 data-driven methods (see Table 4)	5 aggregation functions (see Table 6)			x			x	x
Decerns (1)	Yatsalo et al. (2016)	Value function	Additive (MAVT)					x	x	
Decerns (2)	Yatsalo et al. (2016)	Value function	Additive (MAUT, ProMAA, FMAA, Fuzzy MAVT)	x	x		x	x	x	
D-Sight	D-Sight (2020)	1 data-driven method (min–max)	Additive					x		
GMAA	Insua et al. (2020)	Value function	Additive	x	x		x	x		
Hiview 3	Catalyze (2020)	Value function	Additive					x		
JSMAA	Tervonen (2014)	Value function	Additive	x	x		x	x		
Logical Decisions	Logical-Decisions (2020)	Value function	Additive	x			x	x		
Smart Decisions	Cogentus (2020)	Value function	Additive	x			x	x		
V.I.P	Dias and Climaco (2000)	Value function	Additive		x					
Web-HIPRE	Mustajoki and Hämäläinen (2000)	Value function	Additive					x		
WINPRE	Salo and Hämäläinen (1995)	Value function	Additive	x	x		x	x		

*MAVT* multi-attribute value theory, *MAUT* multi-attribute utility theory, *ProMAA* probabilistic multi-criteria acceptability analysis, *FMAA* fuzzy multicriteria acceptability analysis

**Table 3:** Visualization of output variability in the reviewed software

Software	References	Tabular results				Graphical results				
		Indicators	Indices		Rankings	Indices		Rankings		
		Normalized indicators	Normalized index/indices table	Pairwise confrontation table	Ranking(s) table	Normalized indices with bar/line charts	Range of the indices	Rank frequency matrix (rank frequency)	Bar charts with rank frequency matrix	Rankings comparison with line graph
MCDA Index Tool	This paper	x	x		x	x		x		x
Decerns (1)	Yatsalo et al. (2016)		x		x	x				
Decerns (2)	Yatsalo et al. (2016)							x	x	
D-Sight	D-Sight (2020)		x		x	x				
GMAA	Insua et al. (2020)		x	x	x		x	x	x	
Hiview 3	Catalyze (2020)		x	x		x				
JSMAA	Tervonen (2014)							x	x	
Logical decisions	Logical-Decisions (2020)		x	x		x	x			
Smart decisions	Cogentus (2020)		x	x		x	x			
V.I.P	Dias and Climaco (2000)		x	x			x			
Web-HIPRE	Mustajoki and Hämäläinen (2000)		x			x				
WINPRE	Salo and Hämäläinen (1995)						x			

#### Comparison features

The features used to compare the software were tailored to the capabilities of representing multiple indices and rankings derived from the UAs and SAs. Output variability analysis was thus at the core of the comparison and it was divided in tabular and graphical results.

##### Tabular results

Tabular results are those that are provided in tables and can include:

1. Normalized indicators: according to the chosen normalization method, the indicators have different normalized scales. The possibility of comparing different normalization methods allows to study the effect that each method has on the alternatives with respect to their raw performances;
2. Normalized indices: according to the normalization method(s) and the aggregation function(s), the final score is provided to the user, which will then be used to obtain the ranking(s);
3. Pairwise confrontation table: a comparison table which indicates the (maximum) advantage (difference of index) of each alternative over each other one. It allows to see if and by how much each alternative performs better (or worse) on a pairwise basis.
4. Rankings table: the indices are used to rank the alternatives from the best to the worst. A table (or more, if more than one index is obtained) as in the MCDA Index tool, can thus be used to summarize the results, listing alternatives in a preference-ordered list.

##### Graphical results

Graphical results are those that are provided in the form of graphs and illustrations. They include:

1. Normalized indices with bar/line charts: the index for each alternative is shown in a bar or line chart;
2. Range of the indices: the variability of the indices can be visualized in the range of value for each alternative, with the possibility of ranking the alternatives by input order or by the output one (e.g., minimum value) (Dias and Climaco 2000). This condenses the variability of the indices in an appealing fashion;
3. Rank frequency matrix: it shows the proportion of indices that rank each alternative in a certain position;
4. Bar charts with rank frequency matrix: these charts visualize the rank frequency matrix in bars whose height varies according to the proportion of indices that rank each alternative in a certain position;
5. Rankings comparison with line graph: it allows selecting and comparing the rankings according to the chosen UA and/or SA settings. For example, if multiple aggregation functions are chosen, the user can visualize the impact of changing the function on a ranking from e.g., a fully compensatory (additive) to a very low compensatory degree (harmonic).

#### Results of software comparison

Table 2 summarizes the available normalization methods, aggregation functions, UAs and SAs in the eleven (11) software selected for the comparison. Only two use data-driven normalization, the MCDA Index tool and D-Sight, while all the others implement DM’s-driven normalization, specifically the value function approach.

The first main finding is that the MCDA Index Tool offers many (i.e., eight) options for normalizing the dataset, while all the others allow only the use of one method. As far as aggregation is concerned, all the software implements only a function with a full compensation level. The only exception is the MCDA Index tool, which provides five aggregation functions with variable compensation levels (see details in Sect. 3.3 and Table 6).

UA and SA on preference models can only be performed by the MCDA Index Tool, as all the others implement only one preference model. One exception is Decerns, which provides a value function SA to compare how different shapes of the value functions affect the results. These different functions can actually be interpreted as different normalization strategies (e.g. linear, piece-wise linear, exponential).

Almost all the software (i.e., Decerns, GMAA, JSMAA, Logical Decisions, WINPRE) that support UA on input data accept uncertain performances of indicators as well as of weights. The exceptions are Smart Decisions that only accepts uncertain performances of indicators and V.I.P., which is specifically tailored to the imprecision on weights.

The focus of SA on input data is devoted to the exploration of different sets of weights, as just over 80% of the software (i.e., 9 out of 11; Decerns, D-Sight, GMAA, Hiview 3, JSMAA, Logical Decisions, Smart Decisions, Web-Hipre, WINPRE) are equipped with this capability. Almost 55% of the software (i.e., 6 out of 11; Decerns, GMAA, JSMAA, Logical Decisions, Smart Decisions; WINPRE) also support SA on the input data, with the main feature being the acceptance of uncertain performances on the indicators.

As far as the visualization of the results is concerned, Table 3 provides an overview of their capabilities of visualization of output variability, and well-defined trends can be found. Tabular results are mostly presented using the obtained indices. This is the case in 9 out of the 11 software (i.e., MCDA Index Tool, Decerns, D-Sight, GMAA, Hiview 3, Logical Decisions, Smart Decisions, V.I.P., Web-Hipre, WINPRE). Five of these software (i.e., GMAA, Hiview 3, logical Decisions, Smart Decisions, V.I.P.) also provide a pairwise confrontation table.

This is an interesting feature as it can boost the comparative analysis between alternatives to identify their strengths and weakness. Only the MCDA Index Tool, Decerns, D-Sight, and GMAA explicitly provide the ranking of the alternatives in a table format. The MCDA Index Tool is the only one that uses multiple normalization methods, which justifies it being the only one that provides comparisons of normalized scores.

Graphical results are primarily (63% of the software) based on visualizing the indices using bar and line charts. This is the case for the MCDA Index Tool, Decerns, D-Sight, Hiview 3, Logical Decisions, Smart Decisions, and Web-Hipre. The other solution, used only in cases of multiple indices as output, is to represent them using the range of variation. This feature is provided by GMAA, Logical Decisions, Smart Decisions, V.I.P., and WINPRE.

The visualization of the rankings of the alternatives is less explored when compared to the previous software capabilities. Only four software (i.e., MCDA Index Tool, Decerns, GMAA, and JSMAA) provide a rank frequency matrix, and all these, except the MCDA Index Tool, show such frequencies in the form of bar charts. Lastly, the comparison of the rankings with a line graph is only supported by the MCDA Index Tool.

## Tool description

The MCDA Index Tool^3^ (https://www.mcdaindex.net/) is a web-based software that provides a practical and straightforward guide for the development of indices and rankings. It implements the UA and SA on multiple preference models, which are capabilities not available in any of the reviewed software as discussed in Sect. 2.2 (except for Decerns that supports SA on normalization methods). In particular, it contains a set of steps that can help develop indices by learning and assessing the quality of the outputs. Key features include robustness assessment of the outcomes and a wide range of results’ visualization. The workflow of the tool follows the guidelines for the development of indices described in the literature (Greco et al. 2019; Mazziotta and Pareto 2017; OECD 2008). The user manual for the tool, including the technical details on how to prepare the input data can be found in Zhang et al. (2020). An important assumption that the authors of the tool made is that before using the tool, the user has properly formulated the decision making problem, by developing a dataset with a series of alternatives evaluated according to a coherent set of indicators, as described in several MCDA guidelines (Bouyssou et al. 2006; OECD 2008).

The flowchart of the tool is presented in Fig. 2 and its steps include input data upload, definition of the polarity of the indicators and weighting, choice of normalization method(s) and aggregation function(s), results computation in tabular forms and finally results visualization. Each step is described in detail in the following Sections.

### Input data

**Fig. 1:**
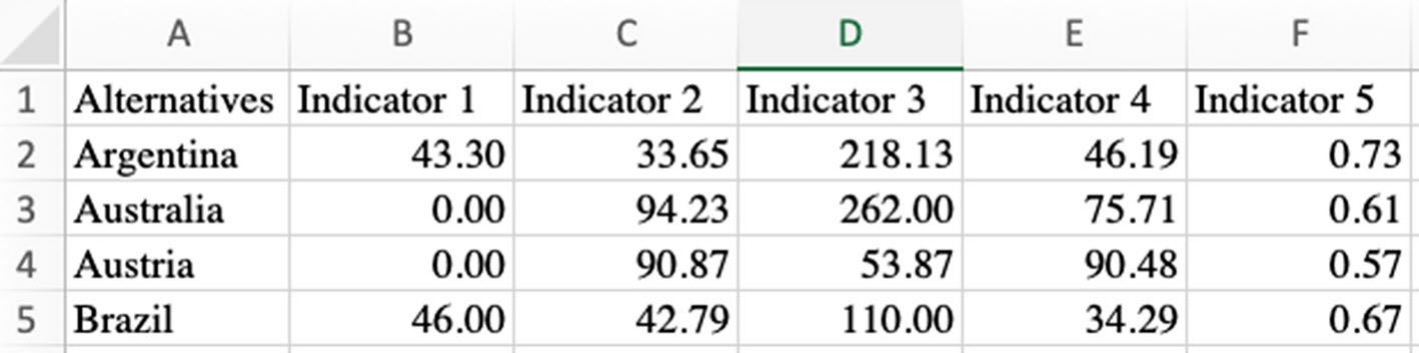
Example of .csv input file

The data of input can be imported in a.csv format. The data file structure resembles a conventional performance matrix with the alternatives listed in the first column, and the indicators in the successive ones. An example is shown in Fig. 1.

### Define settings and weighting

The user has to choose the polarity of each indicator (Mazziotta and Pareto 2017). Positive polarity indicates that the higher the value of the indicator the better, while negative polarity indicates that the lower the value of the indicator the better for the evaluation. It is also possible to explicitly include the measurement units of the indicators, which can be of help during the weighting. The weights of the indicators have to be then chosen. They can be assigned (i) with a simple sliding bar, (ii) by typing them indirectly, or (iii) using the so-called SWING method (Riabacke et al. 2012). The weights are used to define the different priorities of the indicators and they represent the trade-offs that are in place between them. In other words, with respect to pairs of indicators, the weight represents how much the loss on one of them can be compensated with the improvement on the other one.

### Choose the building blocks of the indices

As introduced in Sect. 2, normalization and aggregation are key to define how the input data are made comparable and integrated in the final score. There are several normalization methods available in the literature, and those included in the tool are presented in Table 4, together with a brief description, as well as their pros and cons. The methods based on the ordinal scale (i.e., rank, percentile rank and categorical) only exploit the ordinal character of the input data, making their working procedure quite easy to understand. In addition, they are not affected by the presence of extreme values in the dataset, which can cause skewness in the normalized data.^4^ On the other hand, there is a loss of information between the actual performances, meaning that equal intervals are assumed between consecutive values.

**Table 4:** Normalization methods used in the MCDA Index Tool ( Adapted from Mazziotta and Pareto (2017) and Nardo et al. (2008))

Normalization method		Formula	Description	Pros	Cons
Ordinal scaling	Rank	${I}_{{ic}}={\text{Rank}}\left({x}_{{ic}}\right)$	The alternatives are ranked based on the values of the indicators, from the worst to the best	Easy to understand Not affected by over/under-performers	Loss of information on intervals. It implies that alternatives performing significantly better than others are disadvantaged
	Percentile rank	${I}_{{ic}}=\frac{{a}_{i}+0.5{f}_{{x}_{{ic}}}}{N}$	It uses the percentage of indicators’ values that are equal to or lower than itself		
	Categorical (*a*, *b*, *c*) and (*a*, *b*, *c*, *d*, *e*, *f*)	$${I}_{ic}=\left\{\begin{array}{cc}a& {if x}_{ic}<{x}_{ic=\stackrel{-}{c}}-{\sigma }_{ic=\stackrel{-}{c}}\\ b& {if{x}_{ic=\stackrel{-}{c}}-{\sigma }_{ic=\stackrel{-}{c}}\le x}_{ic}\le {x}_{ic=\stackrel{-}{c}}+{\sigma }_{ic=\stackrel{-}{c}}\\ c& if{x}_{ic=\stackrel{-}{c}}+{\sigma }_{ic=\stackrel{-}{c}}{<x}_{ic}\end{array}\right.$$	It transforms the data according to predefined rules. In the tool, they are driven by the mean and standard deviations of the indicators among all alternatives		
Interval scaling	Standardization (*Z*-score)	${I}_{ic}=\frac{{x}_{ic}-{x}_{ic=\stackrel{-}{c}}}{{\sigma }_{ic=\stackrel{-}{c}}}$	A transformation of the data set with a mean of zero and a standard deviation of one	Preserves the distribution of the values of the indicators	No fixed range of variation Affected by presence and number of over/under-performers^a^
	Min–max	${I}_{{ic}}=\frac{{x}_{{ic}}-\min\left({x}_{i}\right)}{\max\left({x}_{i}\right)-\min\left({x}_{i}\right)}$	It normalizes the data between zero and one using a linear transformation, driven by the minimum and maximum values for each indicator	All range [0–1] or [0–100], easy comparisons Not affected by number of over/under-performers	The ratios are not conserved Affected by presence of over/under-performers^a^
Ratio scaling	Target	${I}_{{ic}}=\frac{{x}_{{ic}}}{\max\left({x}_{i}\right)}$	It normalizes the indicators’ values with respect to the maximum for each indicator	The ratios are conserved Not affected by number of over/under-performers^b^	No fixed range of variation (max is 1) Affected by presence of over/under-performers^a^
Sigmoid scaling	Logistic	${I}_{ic}=\frac{1}{1+{e}^{-\frac{{x}_{ic}-{x}_{ic=\stackrel{-}{c}}}{{\sigma }_{ic=\stackrel{-}{c}}}}}$	It normalizes the data into a sigmoid curve (S-shaped) between 0 (for − inf) and 1 (for + inf)	Reduces the effect of over/under-performers and can avoid trimming	Difficult to explain to non-MCDA experts
	${I}_{ic}$*: *the normalized value of indicator* i* for alternative* c* ${x}_{ic}$*: *the value of indicator* i* for alternative* c* ${a}_{i}$*: *the amount of values lower or equal to itself ${f}_{{x}_{ic}}$*: *the frequency of indicators with same value* x*_*ic*_ *N: *the number of alternatives ${x}_{ic=\stackrel{-}{c}}$*: *the average value of indicator* i* across all alternatives ${\sigma }_{ic=\stackrel{-}{c}}$*: *the standard deviation of indicator* i* across all alternatives $\min\left({x}_{i}\right)$*: *the minimum value of indicator* i* across all alternatives ${\text{m}}{\text{ax}}\left({x}_{i}\right)$*: *the maximum value of indicator* i* across all alternatives		In the presence of over/under-performers, most of the alternatives are normalized to very closely related values^a^		

The other normalization methods consider the information on the quantitative differences of performances. The standardization one provides an overview of how distributed the indicators’ value are from the mean, but does not provide a bounded range of variation, which can be difficult to communicate to stakeholders. Min–max, on the other hand, offers a bounded range the normalized indicators that can enhance comparisons among indicators, at the expense of not maintaining the ratios between the performances and being strongly affected by the presence of outperformers. A method that maintains the ratios of performances between alternatives is the target one, which measures the relative position of a given indicator with respect to a reference point. In this case only the upper limit is fixed and the range is variable. Lastly, a more complex formulation is the logistic method, which reduces the effect of the outperformers by transforming the data into a sigmoid curve (S-shaped) between 0 (for − inf) and 1 (for + inf).

An illustrative example of the effect of normalization functions on raw data is presented in Table 5. The ordinal methods (i.e., rank, percentile, categorical) do not preserve the actual distances between alternatives and they may receive the same normalized value, for the ternary scale in this example. The limited differentiation between performances is one of the disadvantages of using the weakest type of information, as the ordinal methods do. The *Z*-score (standardization) provides an indication of how distant the performances are from the mean, which in this case is 5.33. Min–max has the advantage of providing a bounded range, in this case [0–1], which easily supports relative comparisons. The target method preserves the ratios between the performances, leading to the same average and standard deviation as the raw data. Lastly, the logistic method reduces the effect of top and worst performers, in fact the normalized values are closer together compared to the standardization, min–max and target (linear methods).

**Table 5:**
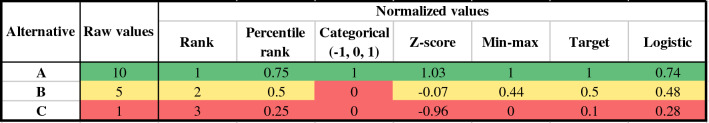
Example to illustrate the effect of different normalization methods (Adapted from Gasser (2019))

3-color scale: the best performance is in green, the worst in red and the one in the middle in yellow

Similarly to normalization, also for the aggregation there is a multitude of available options (Blanco-Mesa et al. 2019). The ones selected for the tool are focused on implementing different levels of compensation between the indicators, as presented in Table 6. As discussed in Sect. 2.1, aggregation functions with different degrees of admissible compensability between performances can be used to consider stakeholders/DMs with different preferences. The selected aggregation functions, in a decreasing compensation order, are additive, geometric, harmonic, and minimum. The median operator is included too, though the compensation level actually varies according to the distribution of the values themselves.

**Table 6:** Aggregation functions used in the MCDA Index Tool (Adapted from Gasser (2019))

Aggregation function	Formula	Level of compensation	Comments
Additive	${\text{score}}_{c}={\sum }_{i=1}^{n}{I}_{ic}\text{ x }{w}_{i}$	Full	Suitable if the decision-makers’ preference values are linear, meaning that the decision-makers accept that the performance of indicators can compensate each other. For example, low-performing indicators can be fully compensated by high-performing indicators
Geometric	${\text{score}}_{c}={\prod }_{i=1}^{n}{{I}_{ic}}^{{w}_{i}}$	Partial	Suitable for decision-makers’ who do not accept full compensation between indicators and want to penalize the alternatives that do perform poorly even on only one This use of this function is not possible if normalized indicators’ values are negative or 0 (lowest performing indicator), as the function cannot be applied. Hence, it is only usable with normalized data sets containing strictly positive numbers
Harmonic	${\text{score}}_{c}=\frac{n}{{\sum }_{i=1}^{n}\frac{{w}_{i}}{{I}_{ic}}}$	Partial (less than geometric)	Same considerations apply as to the geometric. It is even better for more “demanding” decision-makers who desire even less compensation. It is only usable with normalized data sets containing strictly positive numbers
Minimum	${\text{score}}_{c}={\text{min}}\left({I}_{1c},{I}_{2c},..., {I}_{nc}\right)$	None	Particularly suitable if stakeholders want the assessment to be driven by the worst performing indicator
Median	${\text{score}}_{c}= \stackrel{\sim }{I}\left({I}_{1c},{I}_{2c},\dots ,{I}_{nc}\right)$	Depends on the distribution of the indicators’ values	It allows to identify overall trends as one half of an alternative’s indicators are above and the other half below the median Low-performing indicators can be overcompensated by well-performing indicators
	${\text{score}}_{c}$: composite score for alternative* c* ${I}_{ic}$: the normalized value of indicator *i* for alternative* c* ${w}_{i}:$ weight of indicator* i*		*n*: the number of indicators min(): minimum value of all the indicators $\stackrel{\sim }{I}$*: *median of the indicators’ values

Table 7 presents an example of the selected aggregation functions using three alternatives and two normalized indicators, assuming their scale is [0–1]. Alternative A is the best on indicator 1 (i.e., *i*_1_) and it performs very well on it, while its performance is very low and the worst in the set for indicator 2 (i.e., *i*_2_). Alternative C performs average on both indicators, while Alternative B is between the performances of both alternatives. The first notable finding is that due to the full compensation of the additive function, the index for alternative A is the very close to the one of alternative B and C. As the level of compensation gradually decreases from the additive, to the geometric, harmonic and minimum functions, the index of alternative A and B gradually decreases, until reaching its lowest for the minimum function. This phenomenon does not happen for alternative C, as there is no compensation between the performances as the indicators values are the same, showing that a less compensatory DM should prefer this type of alternatives in the dataset. In this example, the median function provides the same results as the additive function because the data set consists of two indicators only.

**Table 7:**
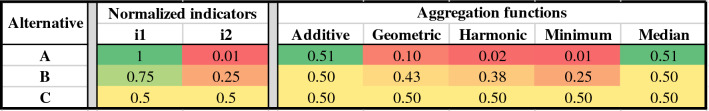
Example to illustrate the effect of different aggregation functions (Adapted from Gasser (2019))

Color scale: the best performance is in green, the worst in red. All other values are coloured proportionally by linear interpolation

Based on the preferences of the involved DM(s), the user can select the normalization methods and aggregation functions to build the indices. After confirming the selection, a combination table is shown to depict the combination of normalization and aggregation that will be used to build the results. In total, 31 combinations are available in the tool, by accounting for multiple compensation levels and approaches to render the indicators on a comparable measurement scale (see Table 8).

**Table 8:** List of the 31 combinations of normalization methods and aggregation functions used in the MCDA Index Tool

	Aggregation function	Normalization method	Comments
1	Additive	Rank	The additive aggregation function is one of the most used. In order to support analysts studying the widest possible variability of the outcomes, it was combined with all types of normalization methods
2		Percentile rank	
3		Standardized	
4		Min–max	
5		Target	
6		Logistic	
7		Categorical (− 1, − 0, − 1)	
8		Categorical (0.1, 0.2, 0.4, 0.6, 0.8, 1)	
9	Geometric	Percentile rank	All the normalization methods were used with the geometric function, except the rank-based one to avoid redundancy with the combination Geometric—Percentile rank (the final scores are almost identical) The treatment of negative and null values was tackled as follows. The standardized data are linearly transformed to positive numbers by adding *x,* i.e., minimum number to make all values positive (“Standardized + *x*”) The range of the min–max normalization method was is modified to [0.1–1] and [0.01–1] instead of [0–1]. The target method was set to a minimum value of 0.1. Finally, the ternary categorical scale was changed to (0.1, 1, 2) and the senary one to (0.1, 0.2, 0.4, 0.6, 0.8, 1)
10		Standardized + *x*	
11		Min–max 0.1–1	
12		Min–max 0.01–1	
13		Target 0.1	
14		Logistic	
15		Categorical (0.1, 1, 2)	
16		Categorical (0.1, 0.2, 0.4, 0.6, 0.8, 1)	
17	Harmonic	Percentile rank	The same normalization methods and the same treatment of negative and null values of the indicators were used for the harmonic function as or the geometric function
18		Standardized + *x*	
19		Min–max 0.1–1	
20		Min–max 0.01–1	
21		Target 0.1	
22		Logistic	
23		Categorical (0.1, 1, 2)	
24		Categorical (0.1, 0.2, 0.4, 0.6, 0.8, 1)	
25	Minimum	Standardized	The minimum function was only applied with the normalization methods that allow a diversification of alternatives based on their worst values. This is only the case for standardized and logistic normalization methods. The others (i.e., rank, percentile rank, min–max, target and categorical) lead alternatives to the same minimum values, providing results that are not useful for a comparative analysis
26		Logistic	
27	Median	Percentile rank	The median function was applied to the percentile rank, standardized, min–max, target and logistic normalization methods The categorical scales were omitted as they lead to pre-defined normalized values, making the methods not suitable for a function that looks at evenly splitting an ordered set The rank normalization method is not included as it is specular to the percentile rank one
28		Standardized	
29		Min–max	
30		Target	
31		Logistic	

### Compute results

**Fig. 2:**
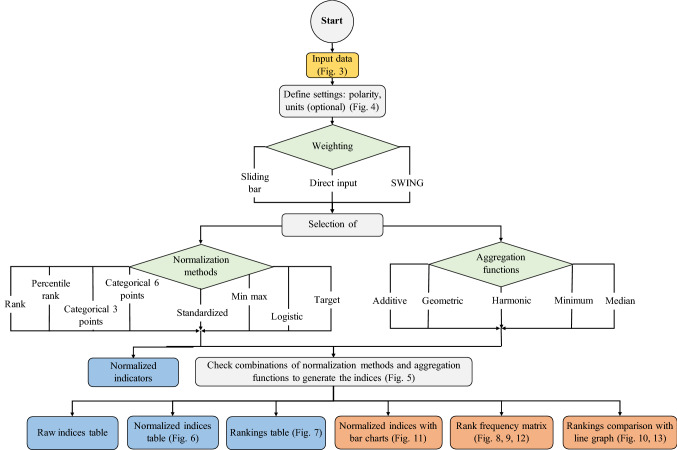
Flowchart of the MCDA Index Tool. Instructions (grey), input (yellow), menu choices (green), tabular results (blue), graphical results (orange)

Once the combinations of normalization(s) and aggregation(s) are confirmed by the user, tabular results are calculated (see Fig. 2). These include the normalized indicators, which allow to directly comparing the alternatives across indicators. In addition, the raw and normalized indices, as well as the rankings are provided.

### Visualize results

The results are also shown in a visual form, in order to enhance the comprehensibility and the comparability between the alternatives (see Fig. 2). The first are indices with bar charts according to the normalization methods or the aggregation functions, empowering a sensitivity analysis on the chosen preference models. The rank frequency matrix, showing the proportion (in %) of indices which rank alternative *x* at the $k$th position allows to study whether a trend in the rankings can be found, and if not, for what reason. Lastly, selected combinations can be chosen by the user to visualize the rankings of interest with a line graph.

## Case study description

This Section presents the application of the MCDA Index Tool to a case study for the assessment of the energy security and sustainability implications of different global energy scenarios. The analysis uses the results generated in the European Union project SECURE (Security of Energy Considering its Uncertainty, Risk and Economic implications)conducted between 2008 and 2010 (Burgherr et al. 2016; Eckle et al. 2011). The choice of this case study is twofold. Firstly, the establishment of a complete dataset for energy scenarios is not trivial, and the SECURE project represents one of the few examples of detailed and realistic energy modeling on a global scale with a large and diversified set of indicators. Secondly, the case study is mostly for demonstration of the tool and for this purpose, it is more pragmatic and informative to use a real-world example that has been extensively analyzed before and is well documented.

In the SECURE project, MCDA was used to comprehensively assess the energy security and sustainability implications of different global energy scenarios, using 13 indicators. However, only one index was used to score and rank the scenarios, resulting from the combination of target normalization and additive weighted sum. The target normalization was chosen as the stakeholders preferred the ratios between the performances to be maintained. The additive weighted sum was used as the aggregation function, assuming that full compensation between the indicators was acceptable. This research aims to explore the application of an extensive robustness assessment of the results by means of an uncertainty and sensitivity analysis on the preference models. The presented MCDA Index Tool has been developed for this specific type of analysis. It is applied to this case study to evaluate its capacity to include and visualize multiple stakeholders’ perspectives, as well as identification of trends in the results. A detailed description on the scenarios formulation as well the selected indicators is available in Eckle et al. (2011). The next sub-sections briefly present the alternatives and the indicators used for this illustrative case study of the tool.

### Alternatives

In the SECURE project, the alternatives are global energy scenarios, which were defined with the Prospective Outlook on Long-term Energy Systems (POLES) model (Checci et al. 2010). POLES allows the identification of scenarios by defining the drivers and constraints for energy development, fuel supply, greenhouse gas emissions, international and end-user prices, from today to 2050. In total, 14 scenarios were analyzed, which consisted of five basic scenarios, and three shock conditions combined with the basic scenarios that resulted in 9 shock scenarios (Checci et al. 2010; Eckle et al. 2011). The basic scenarios included:

- Baseline (BL): development of energy systems until 2050 in the absence of climate policy. Key characteristics of this scenario are that human population grows over nine billion in 2050, global real GDP triples, and global primary energy consumption rises by 70%;
- Muddling through (MT): countries decide to individually manage their energy needs and security, leading to non-coordinated efforts to mitigate climate change. CO_2_ stabilizes to above 500 parts per million by 2100;
- Europe alone (EA): climate policy with target of reducing GHG emissions by 60% in 2050 compared to 1990 levels only in Europe;
- Global regime, full trade (FT): emerging international consensus to tackle climate change leads to agreement of reducing global GHG emissions by 50% compared to 1990 levels. Two sub-scenarios are defined. FT 1: two global markets for CO_2_ (industrialized. vs. developing countries) and FT 2: fully integrated, global market for CO_2_.

The shock scenarios were:

- Nuclear accident (Nuc): due to a nuclear accident the phase-out of existing nuclear plants, with a significant reduction in Europe by 2050;
- Fossil fuel price shock (Sh): increase in the price of oil and gas by a factor of three leads to decrease in their consumption by 10–20% in the short term and an increase in nuclear energy;
- No carbon capture and storage (CCS): the deployment of CSS does not take place due to safety and economic limitations.

The whole set of scenarios considered in the SECURE project is summarized in Table 9. There are a few scenarios that have not been included in the simulation results as they do not substantially differ from other scenarios, and they are indicated with a “–” in Table 9. The first is Nuc shock in EA, which compares to the MT Nuc scenario, with increasing use of fossil fuels to substitute some of the nuclear energy and increasing CO2 emissions on a long-term perspective, despite available CCS technologies. The second is FT with Sh shock, where as a result of a global lower dependence on fossil fuels, the price shock has less impact on long-run demand for oil and gas than in previous scenarios. Lastly, CCS shock in BL is not explicitly shown since CCS plays no role in the Baseline scenario, so this shock does not have an effect on the results.

### Indicators

The sustainability implications and security of supply of the energy scenarios were evaluated with 13 indicators, which included indicators from each of the sustainability pillars, namely environment, economy and society, and from security of supply domain. Table 10 provides a summary of the indicators, together with a brief description, the measurement unit and the polarity. Further details on the indicators can be found in Eckle et al. (2011). Compared to the analysis within SECURE, where four dimensions provided the first level of the hierarchy, and in the case of diversity of resources, severe accidents and oil spills there was a second hierarchy level, in this case study a flat structure of indicators was used. This choice was driven by the current lack of the capability of hierarchical structuring of indicators in the tool, which could be a useful avenue for its further expansion.

**Table 9:** Scenarios developed for the SECURE project (Eckle et al. 2011)

		Basic scenarios			
		Baseline (BL)	Muddling through (MT)	Europe alone (EA)	Global regime—full trade (FT 1 & 2)
Shock events	Nuclear accident (Nuc)	BL Nuc	MT Nuc	–	FT Nuc
	Fossil fuel price Shock (Sh)	BL Sh	MT Sh	EA Sh	–
	No carbon capture & storage (CCS)	–	MT CCS	EA CCS	FT CCS

The basic scenarios are in the second row. The 3 × 4 matrix with the shock scenarios is then the combination of basic scenarios and shock events

**Table 10:** Indicators used to evaluate the scenarios in the SECURE project (Eckle et al. 2011)

Area		Indicators	Description	Unit	Polarity	Weight
Environment	${i}_{1}$	CO_2_ emissions world	Worldwide CO_2_ emissions per capita	t CO_2_/capita	↓	0.17
	${i}_{2}$	CO_2_ emissions EU27	EU 27 CO_2_ emissions per capita	t CO_2_/capita	↓	0.08
Economy	${i}_{3}$	Energy expenditure world	Global energy expenditure per Gross Domestic Product (GDP)	USD/GDP	↓	0.17
	${i}_{4}$	Energy expenditure EU 27	EU 27 energy expenditure per Gross Domestic Product (GDP)	USD/GDP	↓	0.08
Society	${i}_{5}$	Cumulated number of fatalities from accidents	Cumulated number of fatalities from severe (≥ 5 fatalities) accidents in fossil, hydroelectric and nuclear energy chains	Fatalities	↓	0.07
	${i}_{6}$	Fatalities of worst accident	Maximum number of fatalities from severe (≥ 5 fatalities) accidents in fossil, hydroelectric and nuclear energy chains	Fatalities	↓	0.02
	${i}_{7}$	Oil spills risk	Risk of oil spills, proportional to oil used	Mtons	↓	0.04
	${i}_{8}$	Terrorism risk	Number of fatalities based on a cumulated terrorism risk for EU 27, involving a European Pressurized Reactor (EPR), hydropower dam, refinery and Liquified Natural Gas terminal	Fatalities	↓	0.13
Security of supply	${i}_{9}$	Diversity EU27 consumption	Diversity index of EU gross inland energy consumption	Factor 0–1	↑	0.11
	${i}_{10}$	Import independence EU27	Ratio of primary production/gross inland consumption	Factor 0–1	↑	0.11
	${i}_{11}$	Diversity world oil market	Diversity index of net oil exporters from 23 world regions	Factor 0–1	↑	0.01
	${i}_{12}$	Diversity world gas market	Diversity index of net gas exporters from 23 world regions	Factor 0–1	↑	0.01
	${i}_{13}$	Diversity world Coal Market	Diversity index of net coal exporters from 23 world regions	Factor 0–1	↑	0.01

↑: positive polarity = the higher the value of the criterion the better; ↓: negative polarity = the lower the value of the criterion the better

The weights represent trade-offs between the indicators

## MCDA Index Tool in action: application to the SECURE project

The MCDA Index Tool was applied to the SECURE project case study, using the 14 scenarios in Table 9, characterized according to the 13 indicators shown in Table 10. The following Sections describe and illustrate each step of the process of applying the tool.

### Upload input data

**Fig. 3:**
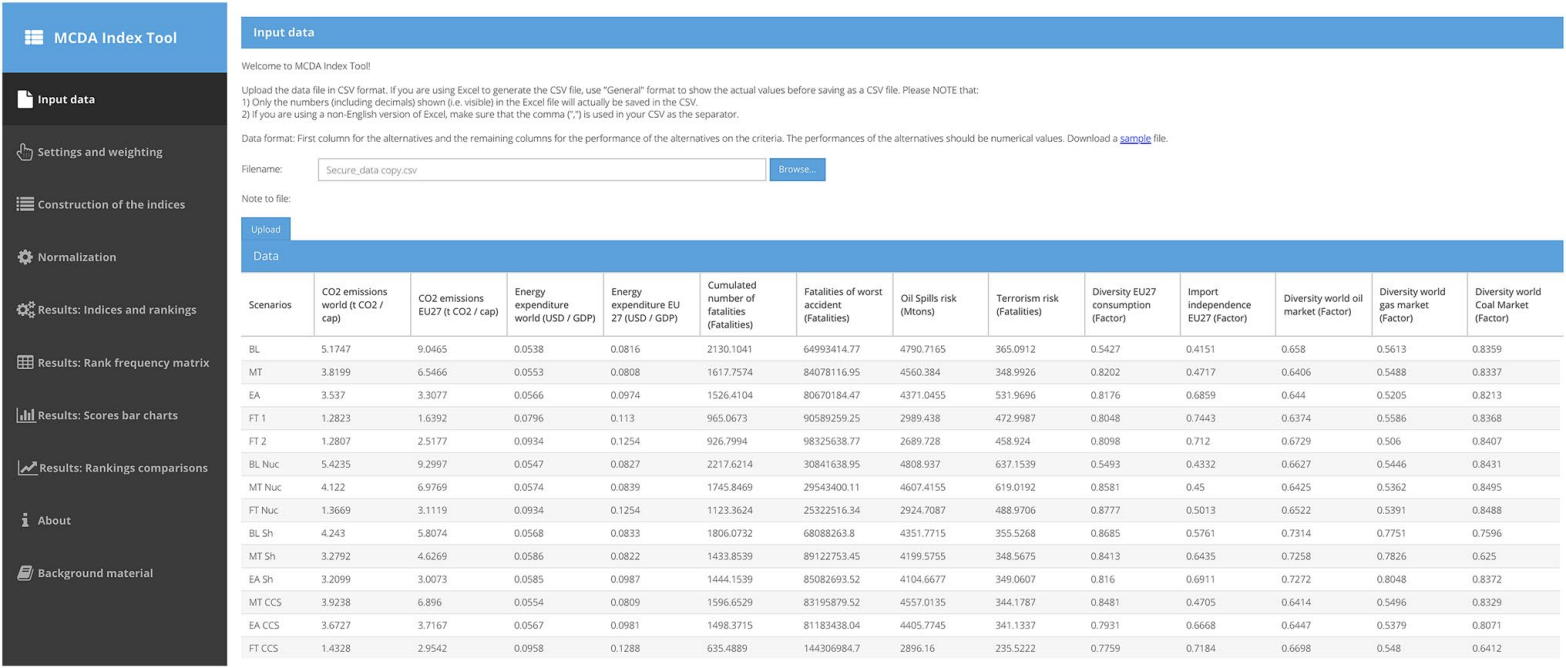
Imported dataset

In the “Input data” page, the.csv file named “Secure_data” was imported for analysis as it is shown in Fig. 3.

### Define settings and weighting

**Fig. 4:**
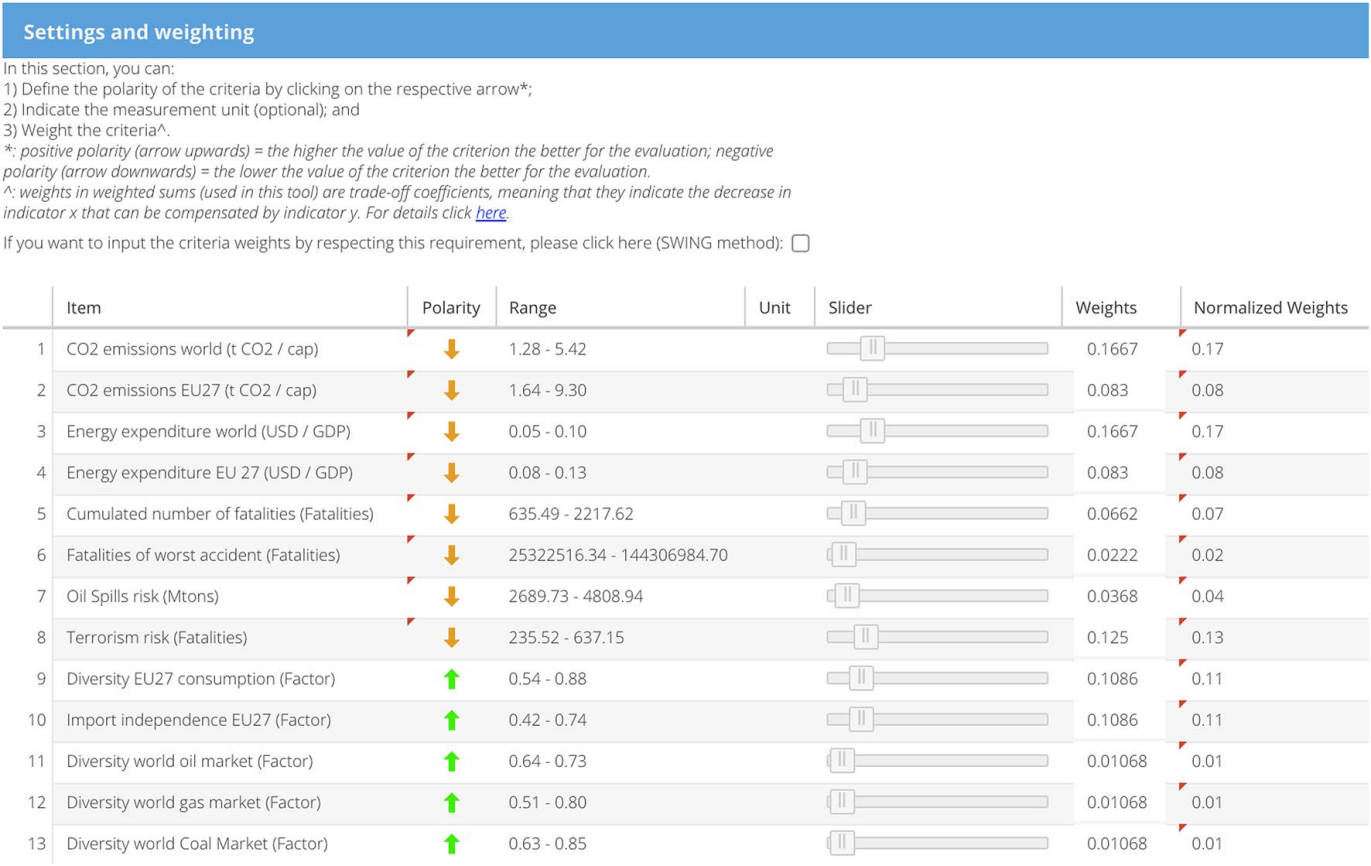
Settings and weighting for the indicators

In the “Settings and weighting” page, the polarity, measurement unit, and weight can be defined for each indicator (see Fig. 4). In this case, a *balanced* weighting profile was selected, which aims to reduce global emissions in the defined scenarios. Therefore, the focus is on worldwide instead of European (EU27) emissions (Eckle et al. 2011). Among the security of supply indicators, those with larger differences between scenarios received higher weights to increase discrimination between scenarios (i.e., Diversity EU27 consumption (${i}_{9}$), Import independence EU27 (${i}_{10}$)). As far as social indicators are concerned, lower weight is given to Fatalities of worst accident (${i}_{6}$) given to the low probability of such an event. Terrorism risk (${i}_{8}$) receives the highest weight in this area, while the remaining ones have weights in between.

### Choose construction of the SECURE indices

The next step of index development consists in (i) the selection of the normalization method(s), which allow transforming all the indicators on the same scale and make them comparable; and (ii) the choice of the aggregation functions, which aggregate all the indicators on the same scale into an index. As mentioned in the Sect. 4, only one normalization method (i.e., target) and one aggregation function (i.e., additive average) were used to develop the index in the SECURE project. For this combination, additional analysis of the weights was conducted to assess when the baseline scenario receives the top rank and, in addition, SA on the weights was performed to explore the effect of different weighting profiles on the rankings (Eckle et al. 2011). However, the influence of different preference models on the results was not explored. Uncertainty and sensitivity analyses have thus been performed to study the variability of the scores and ranking of the SECURE scenarios according to different normalization and aggregation strategies, which is one of the main contributions of this paper.

#### Uncertainty analysis settings

All the admissible combinations of normalization methods and aggregation functions with decreasing compensation level (independent from the indicators’ distributions as in the case of the median) were selected. The minimum operator was also excluded, as it is only driven by the worst value among the indicators and the requirement from the SECURE project was that all the indicators should have contributed to the assessment. This results in 24 combinations, i.e., 24 indices (available in Appendix A of the Electronic Supplementary Information (ESI)). These settings for the UA ensure to consider the combined effect of decision makers accepting compensation between indicators from a complete (with additive aggregation) to a low (with harmonic aggregation) level, as well as ordinal, interval, ratio and sigmoid-based harmonization of the raw indicators.

#### Sensitivity analysis settings

The SA aimed at assessing separately the influence of the uncertainty in the MCDA process, by looking specifically at the effect of the normalization methods and the aggregation functions.

**Fig. 5:**
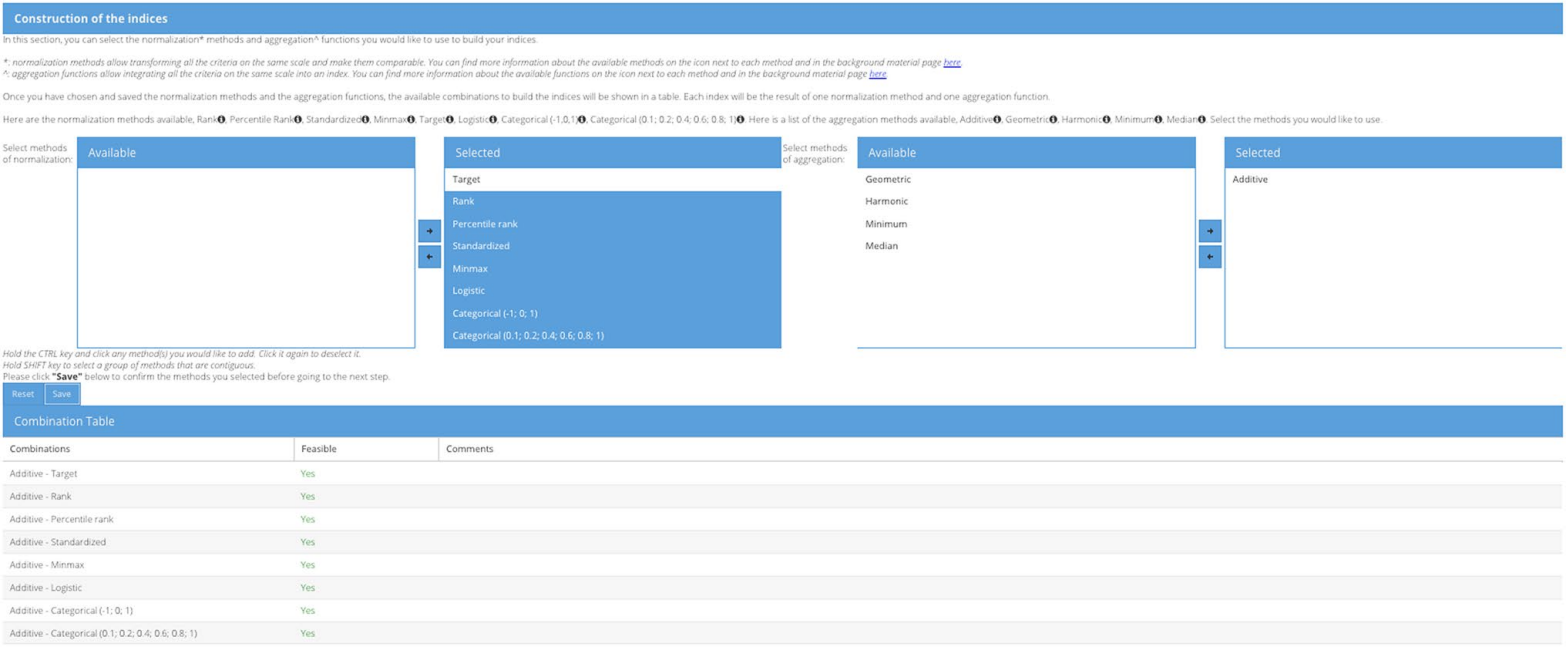
Combinations of normalization methods and aggregation function used in the case study

As far as the normalization methods are concerned, the same aggregation as in the SECURE project (i.e., additive average) is used, combined with all the different normalization methods, resulting in eight combinations (see Fig. 5). This means considering different preferences the DMs/stakeholders could have with respect to how the raw data are made comparable to each other, for example by just considering the ordinal nature of the data (i.e., rank, percentile rank, categorical), the deviation from the mean (i.e., standardized), the distance from the best performer (i.e., target), or by having the same scale range (e.g., min–max).^5^

As far as the aggregation functions are concerned, the same normalization as in the SECURE project (i.e., target) is selected, combined with the three aggregation methods in decreasing compensation level, resulting in three combinations. This allows considering different compensation acceptance of the DMs/stakeholders, from full (i.e., additive), moving to medium (e.g., geometric) until a low level (e.g., harmonic).

### Compute results

**Fig. 6:**
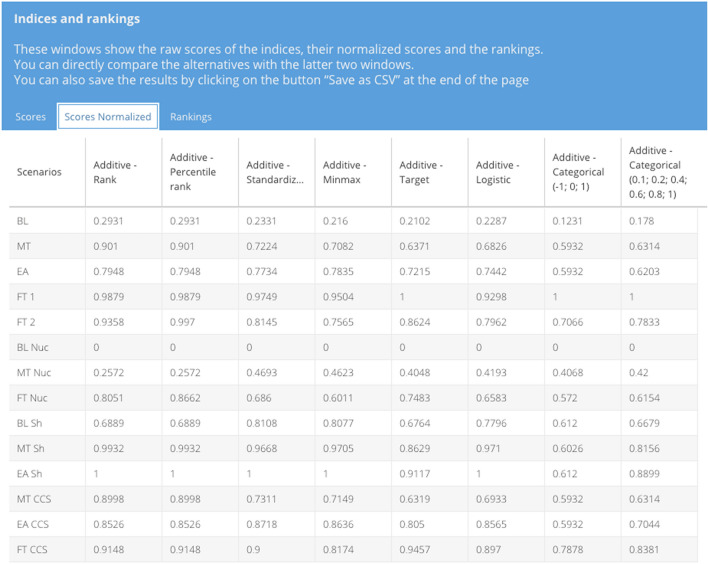
Scores normalized window for the comparison of multiple normalization methods

**Fig. 7:**
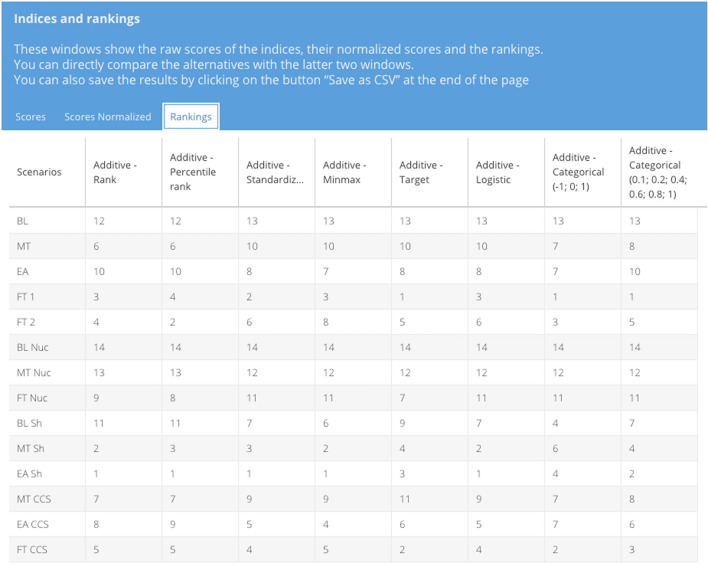
Rankings window for the comparison of multiple normalization methods

Once the combinations are defined, the results are computed by the tool, which provides two main outcomes. The first is the normalized dataset, with one sheet per normalization method selected. The other one includes the indices and rankings. More specifically, this section consists of the raw scores of the indices (named “Scores” in the tool), their normalized scores (named “Scores Normalized” in the tool, see Fig. 6) and the rankings (see Fig. 7). The user can directly compare the alternatives with the latter two tab panels.

### Results: uncertainty analysis

**Fig. 8:**
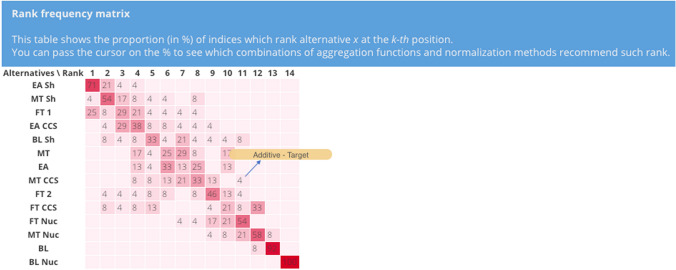
Rank frequency matrix for the comparison of 24 combinations of normalization methods and aggregation functions

The results of UA obtained with different combinations of normalization methods and aggregation functions is plotted by the tool in a rank frequency matrix, as shown in Fig. 8. This figure shows the proportion (in %) of the combinations leading to each rank position. It is the number of the combinations that leads to that specific rank divided by the total number of the combinations. The user can move the cursor on the number in each box to learn which combination(s) rank the alternative under interest at that position. For example, the number 4 in Fig. 8 with the “Additive–Target” yellow box, indicates that the scenario MT CCS is ranked 11th in 4% of the combinations (i.e., one out of 24), which include additive as the aggregation function and target as normalization method.

There are three main findings that can be derived from this UA. The first one is that the worst scenarios, irrespective of the normalization and aggregation, are BL and BL Nuc. BL is ranked second to last in 92% of the combinations, while BL Nuc is always in the last position. This is not unexpected as BL scenario has no climate policy with a global primary energy consumption rise of 70%. In addition, BL Nuc is even worse because with nuclear phase out, the greenhouse gas emissions increase even more, leading it to be the worst performer in five (i.e., ${i}_{1}, {i}_{2}, {i}_{5}, {i}_{7}, {i}_{8}$) out of the 13 indicators. The second finding is that EA Sh ranks robustly within the first four ranks, with a high (i.e., 71%) share of combinations assigning it to the first rank. This trend can be adducted to the low weights assigned to the fatalities-related indicators (i.e., ${i}_{5}$, ${i}_{6}$), where it does not perform as well as on the other indicators compared with the other scenarios. The third result is that in more than 80% of the combinations, there are three scenarios that compete for the first three positions. These are EA Sh, MT Sh, and FT 1. As far as the other scenarios are concerned, the UA shows that their rank can vary considerably according to the combination, and no clear trend can be extrapolated. For example, FT CCS ranking ranges between the 2nd position to the 12th position, while the one for EA CCS is between the 2nd and 9th position. This motivates even more the need to study the effect of the sources of uncertainty on the results, which are analysed in the next section with SA.

### Results: sensitivity analysis

#### Sensitivity analysis on normalization methods

**Fig. 9:**
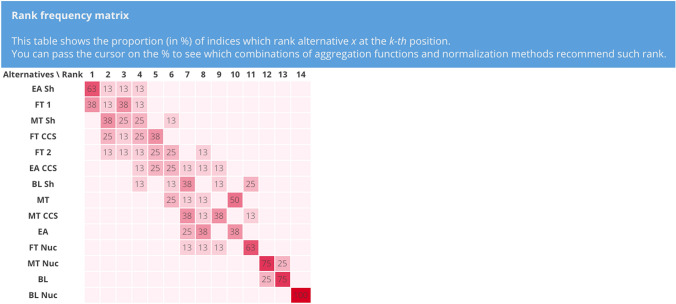
Rank frequency matrix for the comparison of normalization methods

**Fig. 10:**
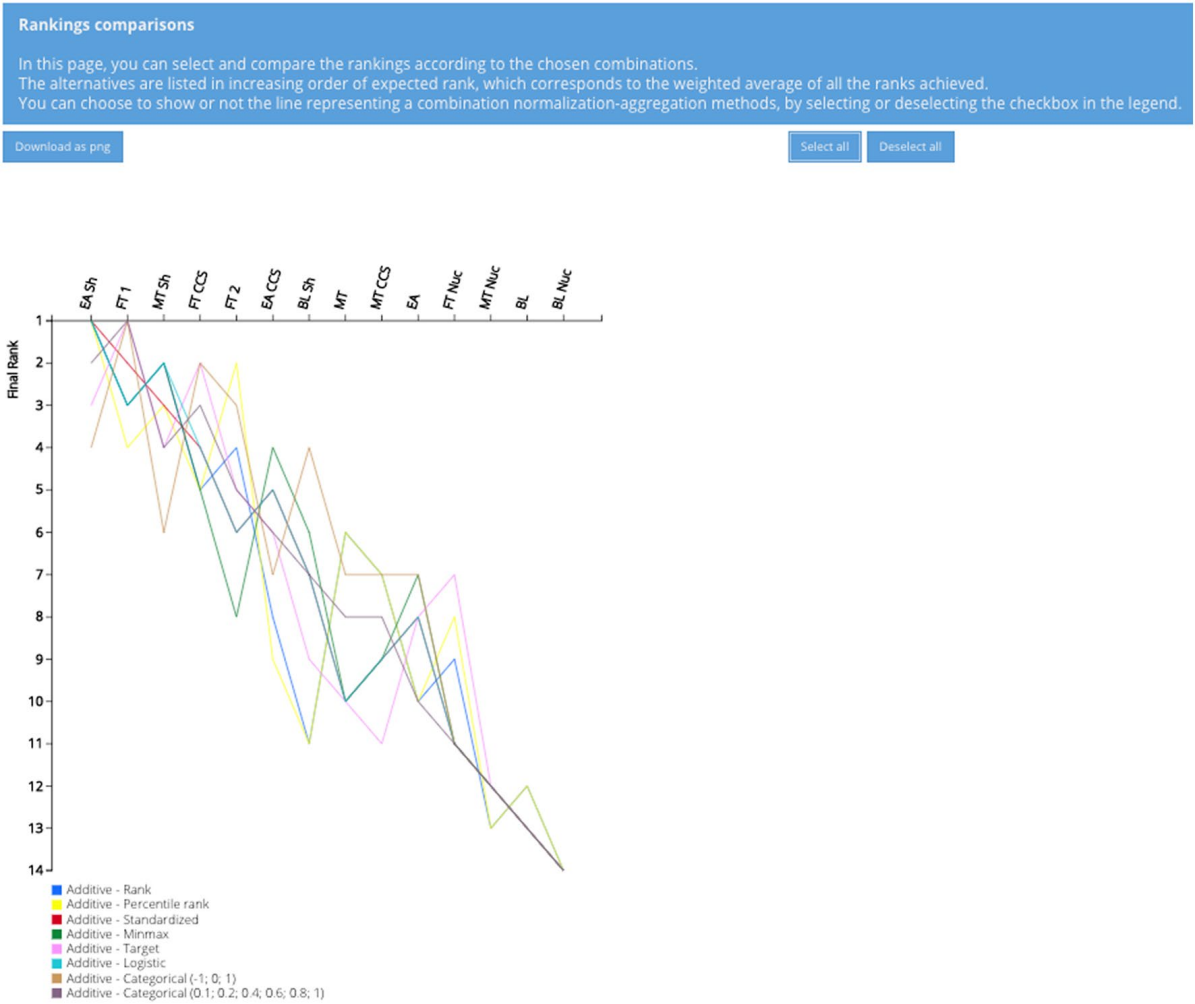
Ranking comparisons of normalization methods with line graph

The results of SA on the normalization methods is represented in a rank frequency matrix and a rankings comparison in Figs. 9 and 10, respectively. Figure 9 shows the share of the combinations that leads to that specific rank with respect to the total number of the combinations (eight in this SA). The same ranking results can also be visualized in another fashion as presented in Fig. 10, by means of a line chart. Based on these two figures, the presence of a trend is visible. EA Sh and FT 1 are never ranked worse than the 4th position, with EA Sh receiving for five out of eight combinations the first rank. The close followers are MT Sh and FT CCS, with a relatively equal share of combinations leading from the 2nd to the 5th (for FT CCS) and 6th position (for MT Sh). BL Nuc emerges as the worst scenario, independently from the type of normalization, whereas MT Nuc and BL consistently rank 12th or 13th and can be considered as robustly poor performers. The remaining scenarios rank variably in the high-middle (4th) to low (11th) positions and there are changes of up to seven ranks, especially for scenarios FT 2, EA CCS, and BL Sh.

When compared to the results for all the combinations (see Fig. 8), it can be seen that FT CCS never ranks lower than 5th (compared to the 12th rank as its worst case in Fig. 8) and FT 2 never ranks worse than 8th (compared to the 11th rank as its worst case in Fig. 8). This finding indirectly indicates that the aggregation function has the most significant effect on the variability of the output for FT CCS and FT 2. On the contrary, EA loses three of its best ranks, and MT, BL Sh, and EA CCS each lose two, compared to their best case with all the combinations (see Fig. 8).

#### Sensitivity analysis on aggregation functions

**Fig. 11:**
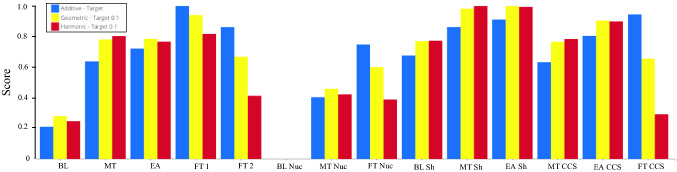
Scores normalized window for the comparison of aggregation functions

The tool provides a bar graph comparing the normalized indices according to the selected normalizations and aggregations. Figure 11 shows the normalized scores of the index for each scenario according to different compensatory algorithms. It is clearly visible how FT 1, MT Sh and EA Sh consistently score well in all the three aggregations, while BL Nuc is always performing very poorly. In addition, there are some scenarios like FT 2, FT Nuc and FT CCS, which are considerably penalized by a decreasing level of compensation, since they lose more than half of their score as the compensatory degree of the aggregation algorithm lowers. This is especially caused by their low relative performance on ${i}_{3}=$ Energy expenditure world and ${i}_{4}=$ Energy expenditure EU 27.

**Fig. 12:**
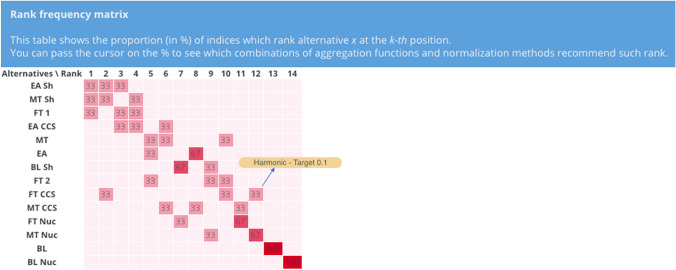
Rank frequency matrix for the comparison of aggregation functions. Note the yellow box indicating which combination of normalization (i.e., target method) and aggregation (i.e., harmonic function) assigns FT CCS to the 12th rank

**Fig. 13:**
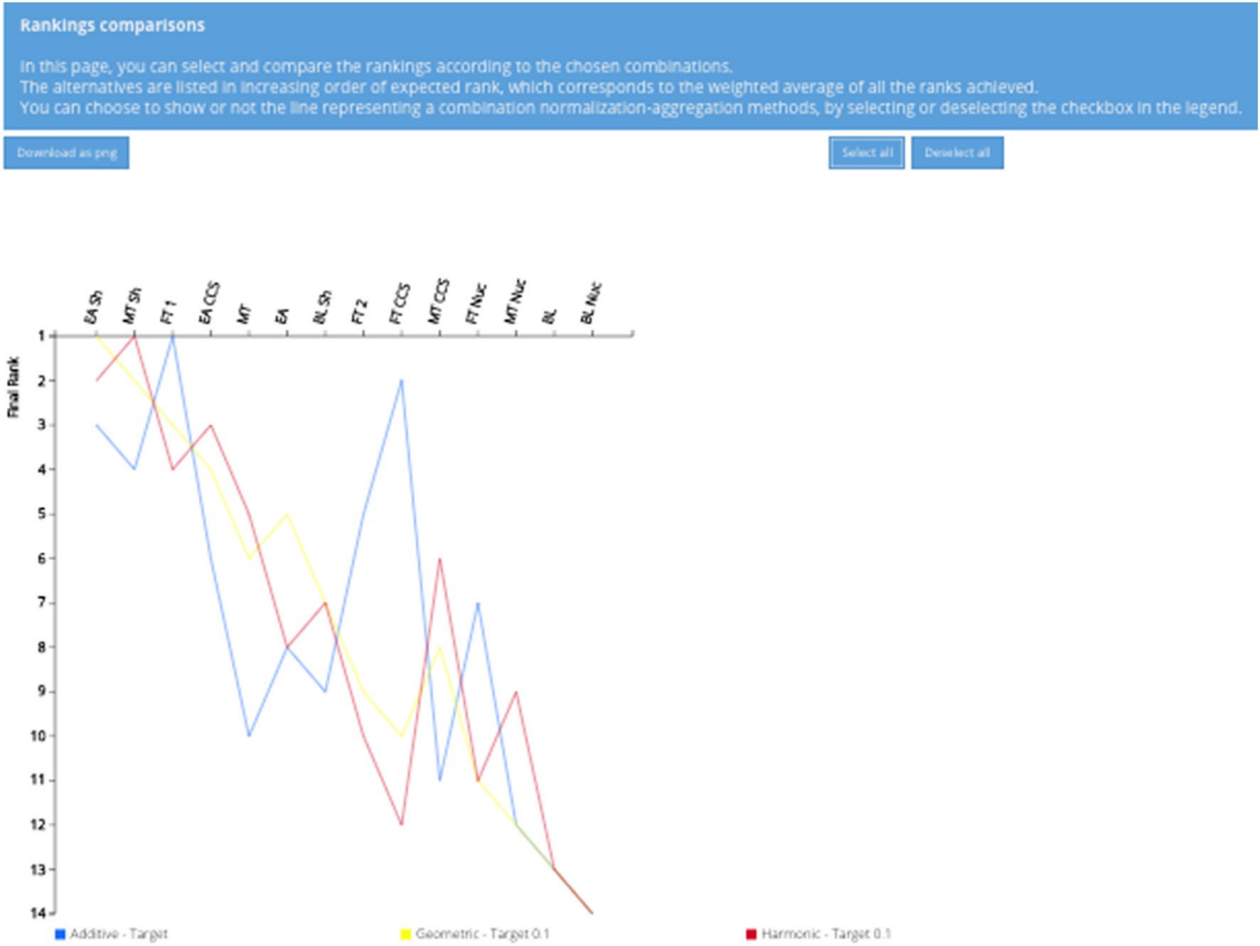
Ranking comparisons of aggregation functions with line graph

Similarly to the previous set of combinations, also those driven by different aggregation functions can be used to study the variability of the rankings, as Figs. 12 and 13 confirm. They provide a complementary display of the findings, using the ranks of the scenarios instead of their scores. Interestingly, the first three scenarios are the same as those found for the comparison of combinations of normalization methods, namely EA Sh, MT Sh and FT 1. In this case as well, EA Sh is one of the best performers, with the additive, geometric and harmonic functions assigning it to the 3rd, 1st and 2nd rank, respectively. MT Sh still performs relatively well, as the additive, geometric and harmonic place it in the 4th, 2nd and 1st rank, respectively. It is evident that BL Nuc is still the worst performer, with all the combinations placing it in the last position. BL and MT Nuc are also ranked in the lower part of the graph as it was the case when using different normalization methods. These results also emphasize a large rank variability especially for FT CCS (from 2nd to 10th and 12th), as well as still notable rank changes up to five positions for FT 2, EA, MT and MT CCS. As reported above, the low performance on even only one indicator causes a remarkable penalization for the scenarios, which is especially evident for FT CCS.

## Discussion

This paper shows the added value of the implementation of a web-based software, called MCDA Index Tool, to perform in a single place the upload of performance data on a set of discrete alternatives, selection of weighting of indicators, choice of normalization methods and aggregation functions, and calculation and visualization of indices and rankings. The analyst can learn and perform in a single system all the steps that he/she needs to follow once a problem is framed and a set of alternatives should be evaluated comprehensively with an index, based on a set of indicators. The main advantage of this tool is that it empowers the user to perform uncertainty and sensitivity analysis on two key choices during the development of a CI, the normalization and the aggregation stages. With these features, the user can study the variability on the results, especially in decision contexts that involve more than one DM/stakeholder, having different requirements on how to normalize and/or aggregate the results into an index. These UAs and SAs on preference models can be particularly useful in MCDA problems where there is no clear DM and a multitude of perspectives should be accounted for, like for example in Life Cycle Assessment (Dias et al. 2019). In this case, decisions must be taken while accounting for preferences of stakeholders with divergent compensatory attitudes. For example, full compensation from the industry clearly contrasts with very limited compensation from the regulatory or environmental interest groups. Another example where the different levels of compensation can have a crucial role, especially in the interpretation and aggregation stage, is the one of Sustainable Development Goals (SDGs) (Muff et al. 2017). In this case, there are explicit trade-offs that must be discussed and different policymakers can have different perspectives on how much one goal can compensate another, which directly affects the suitable aggregation function. These UAs and SAs could clarify how variable the ranking of the alternatives can be when explicitly including these divergent perspectives.

This is not a tool to find the “best” approach in general to develop an index and rank alternatives. It is rather a platform that can be used to explore the impact that different strategies to normalize the raw dataset as well as to aggregate such normalized information can have on the final outcome, in this case a comprehensive score of performance of the alternatives, and consequently on their ranking. It can also be used to develop combinations of normalization and aggregation strategies that satisfy the requirements of the decision makers/stakeholders. For example, if they prefer to have a normalized dataset with the same range and they desire very low compensation between indicators and want to penalize the alternatives that do perform poorly even on only one criterion, then the combination with min–max normalization and the harmonic aggregation can be used.

The tool was used to test the robustness of the model developed as part of the EU project SECURE, where only one normalization and aggregation was used, which led to a single score, hence only one ranking of the alternatives (i.e., scenarios) (Eckle et al. 2011). More specifically, only a single strategy to normalize the data was chosen and a full compensatory algorithm selected. This raises the question on the stability of the results in case of changes with respect to how the raw data are made comparable (i.e., at the normalization stage) and then aggregated to provide an overall score. This type of research question can be addressed by the present tool, by conducting a robustness assessment of the scoring and ranking of the scenarios, using uncertainty and sensitivity analysis on the normalization methods and aggregation functions. This analysis confirmed that the best scenarios include EA Sh, FT 1 and MT Sh, as in the SECURE project. Based on the UA results, these scenarios consistently rank in the first four positions in more than 80% of the combinations of normalizations and aggregations. One discrepancy with the findings in the original SECURE analysis is that FT 2 does not appear among the best scenarios and it is confined to the middle ranks, possibly because of its relatively worse performance on a few key indicators, like global and EU energy expenditures (${i}_{6}$ and ${i}_{7}$). As far as impact of the aggregation function is concerned, most of the results variability can be seen in scenarios FT CCS (from 2nd to 12th rank), FT 2 (from 5 to 10th rank), FT Nuc (from 7 to 11th rank), and MT (from 5 to 10th rank) as Figs. 12 and 13 show. The latter results show how the different degrees of compensation of the aggregation functions can drastically influence the results for some scenarios. This is particularly remarkable for FT CCS, because it was ranked second in the SECURE project, while in this research it is found that it could be ranked as low as 12th if a less compensatory decision maker/stakeholder is accounted for. The worst performers were confirmed with all modeling settings, with BL Nuc as the worst one, preceded by BL and MT Nuc, which coincide with the results from SECURE.

The added value of the tool is that it allows to dynamically visualize the changes in the scores/ranking of the alternatives and understand how:

1. the comparison of the performances between alternatives can be exploited in different forms (only ordinal or cardinal differences);
2. the compensatory attitude of the DM can affect the results.

This shows (i) robustly ranked scenarios for which even a considerable variability of comparison of performances/compensatory attitude does not affect the rankings considerably (i.e., by a few positions like for EA Sh, BL, BL Nuc) and (ii) how unstable scenarios can be identified, like FT CCS, FT 2, FT Nuc, BL Sh, EA and MT.

Compared to the single score evaluation, the tool allows to discuss the implications that the preferences of different decision makers can have on the final scoring/ranking. The tool allows to also identify the most unstable alternatives, those that require further scrutiny and discussion as their overall performance can change considerably according to the desired modeling preferences of the DMs/stakeholders. From a complementary perspective, the tool allows starting with the widest uncertainty in the problem formulation and then, possibly according to the DMs/stakeholders’ input, the preference models that are not realistic can be deleted.

The tool supports dynamic analysis of the results with the rank frequency matrix, especially by means of the yellow box indicating which combinations lead to a certain rank (see Fig. 12). In fact, this is a valuable feature that can be used to support discussions with the DMs, adding a layer of transparency to the final results. In addition, the rankings comparisons with the line graph let the analyst compare the outcomes of the combinations of interest, which can help reaching a final decision on the preferred alternative(s), according to the preferences of the DMs/stakeholders. In the case study, this could involve individual scenario variants, for example for the baseline scenarios or the scenarios with the same shock events.

Finally, the output variability must also be contextualized with respect to the given weighting profile. In other words, the tool allows identifying and visualizing relevant patterns, which then need to be interpreted by the analyst (or DM if he is capable of). It is important to fruitfully use the outputs to help devising recommendations for implementation. A key contribution of the tool in this regard is that it can visually facilitate this process, so that it is more understandable. For example, in case the DMs/stakeholders have a low compensatory attitude and are particularly interested in possibly implementing scenarios within a CCS shock event, then they should assess whether there could be measures to improve their performance with respect to the fatalities of the worst accident (${i}_{6}$) and diversity of resources indicators (i.e., ${i}_{11}$, ${i}_{12}$, ${i}_{13}$).

## Conclusions

The MCDA Index Tool (https://www.mcdaindex.net/) differs from the other MCDA software in that it includes several normalization methods and aggregation functions and provides the possibility of combining them to develop indices and consequently rank alternatives in a comprehensive framework. The structure of the tool allows a dynamic development of the index, including the upload of the raw data, the selection of the weights, and the choice of the normalization and aggregation strategies. The proposed tool can be used by decision analyst as an exploratory strategy during the MCDA process, aiding high-level DMs and stakeholders to explore the implications that different strategies to develop the CI can have on the results. A key advantage of the tool is that it empowers the user to study output variability of the index by performing uncertainty and sensitivity analysis on the preference models. This includes varying the harmonization method used to normalize the data (including ordinal, interval, ratio, and sigmoid) and the aggregation operator (from a full to null compensatory attitude) used to aggregate the normalized indicators in a single score. Another notable contribution of this tool consists in the visualization of the results, from the scores and the rankings in a tabular form to the comparison of the normalization or aggregation approaches on bar charts. Furthermore, the rankings are widely explored with rank frequency matrices and rankings line charts, so that the user can clearly assess the robustness of the results, understand which combinations cause the wideset variability in the results and further investigate combinations of interest that the decision-makers/stakeholders might be mostly interested in. All these functionalities are provided in a unique web-based software, which can help analysts developing indices while learning about the implications behind the choices of certain normalization and aggregation strategies, and dynamically assessing the changes that these choices have on the results.

The DMs and stakeholders that are involved in MCDA processes are normally experts that know the problem well and also understand it (at least to some degree), but they are (usually) not familiar with MCDA from a mathematical standpoint. The MCDA Index Tool provides them with a tool to supplement their often-heuristic approaches with a formal set of decision analysis instruments. In this way, it can support the so-called “formal models” of heuristics (e.g., Mousavi and Gigerenzer (2017)) compared to “informal models” of heuristics (e.g., Kahneman and Tversky (1982)).

A case study with data from the EU project SECURE was used to show all the five steps of the tool, conducting a detailed uncertainty and sensitivity analysis for 14 energy scenarios by accounting for different data normalization strategies and compensatory attitudes of the decision makers/stakeholders. It was confirmed that most of the best and worst scenarios proposed in the SECURE project are stable in their respective performance ranges. However, there are a few exceptions, indicating that some scenarios can receive a very different rank (with up to 10 rank differences), while varying the compensatory attitude of the decision makers/stakeholders. This finding confirms the usefulness of the tool to test the stability of rankings driven by a single score.

There are promising research avenues that can be pursued to expand the decision support capabilities of the proposed tool. Firstly, the possibility of including a hierarchical structure of the indicators, instead of only a flat one, would allow to formulate problems with a large number of indicators in a more well-organized format (Corrente et al. 2013). Inspiration can be taken from some of the reviewed software including Decerns, Smart Decisions and Web-HIPRE. The inclusion of the capacity to use uncertain performances and or weights would allow to model multiple preference models with a further uncertainty management component (Pelissari et al. 2018). Logical decisions can provide several options to consider for such a purpose. It could also be interesting to embed a global sensitivity analysis package like the one proposed by Lindén et al. (2018) to evaluate the implicit influence of each indicator driven by the correlation structure. Furthermore, a summary measure indicating the change of rankings for the same alternatives according to different combinations could be also used in the tool to enhance the high-level assessment of model stability. Several of these possible solutions are presented for example in Kadziński and Michalski (2016). Lastly, aggregation methods that still provide an index could be integrated to account, for example, for the interactions between the indicators, such as with the Choquet integral (Grabisch and Labreuche 2016).

CIs are developed to evaluate multi-dimensional concepts, for which there is not usually a measure to be used to assess how “right” or “wrong” their outcome is. The assessment of a CI is mostly related to the transparency and reproducibility of the process used to develop it (Bouyssou et al. 2002, 2015; Greco et al. 2019; Nardo et al. 2008). With this tool, the authors think that some support is provided to the user in that direction, with a key focus on the normalization and aggregation steps.

## Electronic supplementary material

Below is the link to the electronic supplementary material.

Supplementary file 1 (DOCX 145 kb)

## Footnotes

1 Score, index and Composite Indicator (CI) are used interchangeably in the paper. They refer to the single outcome obtained from the aggregation of the indicators.

2 It is assumed that the user of the MCDA Index Tool has already conducted all these steps when starting to work with the tool.

3 From now onwards, the term “tool” is referred to the “MCDA Index Tool”.

4 These values are usually called outperformers or outliers. They are extreme performances of the indicators when compared to the other values for the same indicator (Ghasemi and Zahediasl 2012).

5 It must be noted that the use of different normalization methods implies that different trade-offs between the indicators are accepted (Gasser et al. 2020), which is the case in this case study. This can actually be also seen as a sensitivity analysis on the variability of the implicit trade-offs between the indicators. Furthermore, when using ordinal normalizations, the information of the extent of differences between the performances is lost, i.e. not taken into account (OECD 2008).
