# Supplementary material for: MCDA Index Tool: an interactive software to develop indices and rankings
Source: Environ Syst Decis. 2020 Jul 16;41(1):82–109. doi: 10.1007/s10669-020-09784-x (PMC7365520; doi:10.1007/s10669-020-09784-x)

**Electronic supplementary information (ESI)**

MCDA Index Tool - An Interactive Software to Develop Indices and Rankings

Marco Cinelli^1,2,^,*^, Matteo Spada^3^, Wansub Kim^1^, Yiwen Zhang^1^, Peter Burgherr^3^

*^1^ Future Resilient Systems (FRS), Swiss Federal Institute of Technology (ETH) Zürich, Singapore-ETH Centre (SEC), Singapore*

*^2^ Institute of Computing Science, Poznań University of Technology, Poznań, Poland*

*^3^ Paul Scherrer Institut (PSI), Laboratory for Energy Systems Analysis, Villigen PSI, Switzerland*

^ Current address: Environmental Decision Analytics Branch, Land Remediation and Technology Division, Center for Environmental Solutions and Emergency Response, Office or Research and Development, U.S. Environmental Protection Agency, Cincinnati, Ohio, USA

^*^ Corresponding author: email: [marco.cinelli@put.poznan.pl](mailto:marco.cinelli@put.poznan.pl)

Table of Contents

[Appendix A – 24 combinations of normalization methods and aggregation functions used in the SECURE project uncertainty analysis 2](#_Toc40620331)

## Appendix A – 24 combinations of normalization methods and aggregation functions used in the SECURE project uncertainty analysis


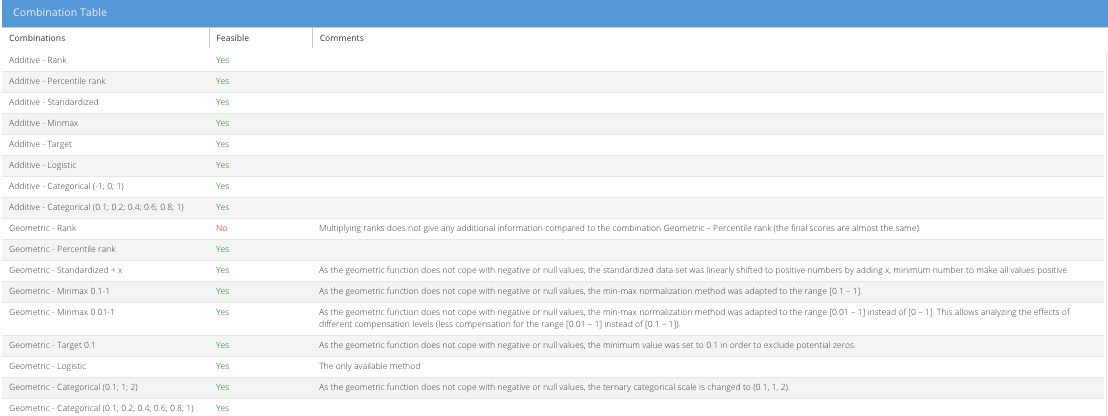


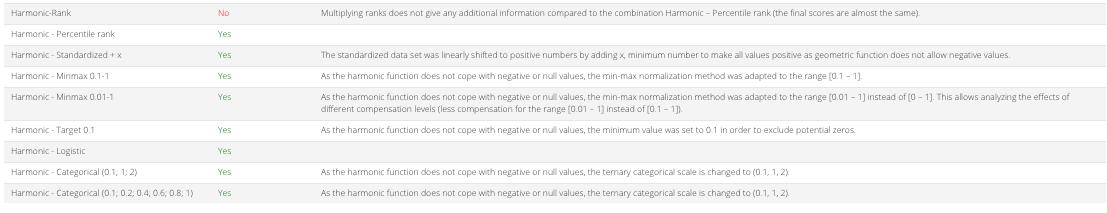

Supplement: Supplementary file 1 — (DOCX 145 kb) [file 10669_2020_9784_MOESM1_ESM.docx]
